# Elastic Tethers Between Separating Anaphase Chromosomes Regulate the Poleward Speeds of the Attached Chromosomes in Crane-Fly Spermatocytes

**DOI:** 10.3389/fmolb.2020.00161

**Published:** 2020-07-29

**Authors:** Arthur Forer, Michael W. Berns

**Affiliations:** ^1^Biology Department, York University, North York, ON, Canada; ^2^Department of Surgery, Biomedical Engineering and Developmental and Cell Biology, Beckman Laser Institute, University of California, Irvine, Irvine, CA, United States; ^3^Department of Bioengineering, Institute for Engineering in Medicine, University of California, San Diego, San Diego, CA, United States

**Keywords:** inter-chromosome signals, spindle forces, chromosome movements with severed microtubules, elastic tethers connect chromosomes, crane-fly spermatocytes, cutting chromosome arms, telomeres

## Abstract

Elastic “tethers” connect separating anaphase chromosomes in most (or all) animal cells. We tested whether tethers are involved in coordinating movements of separating anaphase chromosomes in crane-fly spermatocytes. In these cells the coupled movements of separating chromosomes become uncoupled after the tethers are severed by laser microbeam irradiation of the interzone region between the chromosomes (Sheykhani et al., 2017). While this strongly suggests that tethers are involved with coordinating the poleward chromosome movements, the experiments are open to another interpretation: laser irradiations that cut the tethers also might damage something else in the interzone, and those non-tether components might regulate chromosome movements. In the experiments reported herein we distinguish between those two possibilities by disabling the tethers without cutting the interzone. We cut the arms from individual chromosomes, thereby severing the mechanical connection between separating chromosomes, disconnecting them, without damaging components in the interzone. Disabling tethers in this way uncoupled the movements of the separating chromosomes. We thus conclude that tethers are involved in regulating the speeds of separating anaphase chromosomes in crane-fly spermatocytes.

## Introduction

This article deals with possible functions of “tethers,” elastic connections between anaphase chromosomes. Tethers extend between the telomeres of all pairs of separating anaphase chromosomes in all (or most) animal cell spindles (Forer et al., 2017; Paliulis and Forer, 2018). One cannot identify tethers using phase-contrast or DIC microscopy, however. Their presence is deduced by severing chromosome arms during anaphase, after which the resultant arm fragments move across the equator, led by their telomeres, until they reach their partner telomeres (LaFountain et al., 2002; Sheykhani et al., 2017). Cutting the tethers, i.e., cutting with a laser between an arm fragment and the telomere to which it is moving, stops the movements of the arm fragments (Sheykhani et al., 2017). Arm-fragment movements are not caused by microtubules because the movements require both telomeres, the telomere on the arm fragment and the telomere on the partner chromosome to which the arm fragment moves: if either telomere is laser ablated the fragment does not move (LaFountain et al., 2002). Further, when the chromosome fragment is cut in half only the part with the telomere moves (LaFountain et al., 2002). Yet further, arm fragments produced in taxol-treated cells move at the same speeds as in normal cells (Forer et al., 2018) even though taxol stabilises spindle microtubules and blocks spindle transport of akinetic material (LaFountain et al., 2001). Thus, arm-fragment movements are not due to microtubules and seem to require connection between telomeres. Those connections are not from ultra-fine DNA threads. Tethers connect every anaphase chromosome pair, but ultra-fine DNA threads connect only a fraction of anaphase chromosomes, and only some of those connections are at telomeres (e.g., Chan et al., 2007; Barefield and Karlseder, 2012; Gemble et al., 2015). Further, ultra-fine DNA threads are not elastic. They slow anaphase chromosomes (Su et al., 2016) whereas tethers do not: anaphase velocities are unchanged when tethers are cut (Sheykhani et al., 2017; Forer et al., 2018). Except for data indicating that tethers are not microtubules or ultra-fine DNA bridges, the composition of tethers is not known. However, we and others (Fabian et al., 2007a, b; Forer et al., 2017; Sheykhani et al., 2017) have speculated that perhaps tethers contain the elastic muscle protein titin, since titin extends between the arms of separating anaphase chromosomes in crane-fly spermatocytes (Fabian et al., 2007a, b). Myosin also extends between the separating telomeres, but neither myosin inhibitors nor actin inhibitors block backwards movements of telomeres towards each other (Fabian et al., 2007a).

Tethers are universally present in animal cells, connecting all separating chromosome pairs (Paliulis and Forer, 2018). They also seem to be present in plant cells, as judged by stained connections between anaphase telomeres (Cleland, 1926), though there have been no experiments on plant cells to see if arm fragments move. While all separating anaphase chromosomes are connected by tethers, not all arms are (Forer et al., 2017). In crane-fly spermatocytes in particular, the cell studied in the present experiments, each pair of separating anaphase autosomes is connected by tethers but only two of the four trailing arms are connected, as determined by severing several arms on the same chromosome (LaFountain et al., 2002; Forer et al., 2017; Sheykhani et al., 2017. Also see Adames and Forer, 1996; Forer and Pickett-Heaps, 1998). Tethers do not maintain their elasticity as they elongate: as the tether length (the distance between telomeres of separating chromosomes) increases, arm fragments move slower, move only part way to the partner, or, with yet longer tethers, do not move (LaFountain et al., 2002; Forer et al., 2017). The absence of fragment movement with long tethers is because tethers lose elasticity as they elongate. We know this because the two separating chromosome arms each contract in length (by about 10%) when tethers are severed, and they contract even at tether lengths that are so long that the arm fragments do not move (Forer et al., 2017). It may be that dephosphorylation of tethers causes them to lose elasticity (Kite and Forer, 2020) just as dephosphorylation of muscle titin in the PEVK region causes titin to lose elasticity (Hidalgo et al., 2009; Kõtter et al., 2013; Hamdani et al., 2017).

Do tethers coordinate the movements of separating anaphase chromosomes? Evidence that movements of separating chromosomes in crane-fly spermatocytes are coordinated (coupled, linked) derives from non-laser ultraviolet-light (UV) microbeam irradiations of individual kinetochore spindle fibres. Those studies used as UV source a high-pressure mercury-arc lamp that emits a broad spectrum of wavelengths restricted by a monochromator to a peak wavelength (± half-width) at a given setting (Forer, 1991). Irradiation of a 1–2 μm spot on a kinetochore spindle fibre causes different effects with different wavelengths. When individual anaphase kinetochore spindle fibres were irradiated with a UV microbeam of wavelength 260 or 280 nm, the kinetochore microtubules were severed and the associated chromosomes generally continued to move (Sillers and Forer, 1983; Forer, 1988; Forer and Wilson, 1994; Spurck et al., 1997; Forer et al., 2003). At wavelengths 270 or 290 nm, the chromosome associated with the irradiated kinetochore spindle fibre generally stopped moving (Sillers and Forer, 1981b), independent of whether the kinetochore microtubules were severed (Sillers and Forer, 1983; Forer, 1988). Thus, UV microbeam irradiations of anaphase kinetochore spindle fibres can sever microtubules and they can stop chromosome movements, depending on the UV wavelength, effects which occur independently of each other. These experiments also indicated that chromosome movements are coupled, as follows.

**FIGURE 1:**
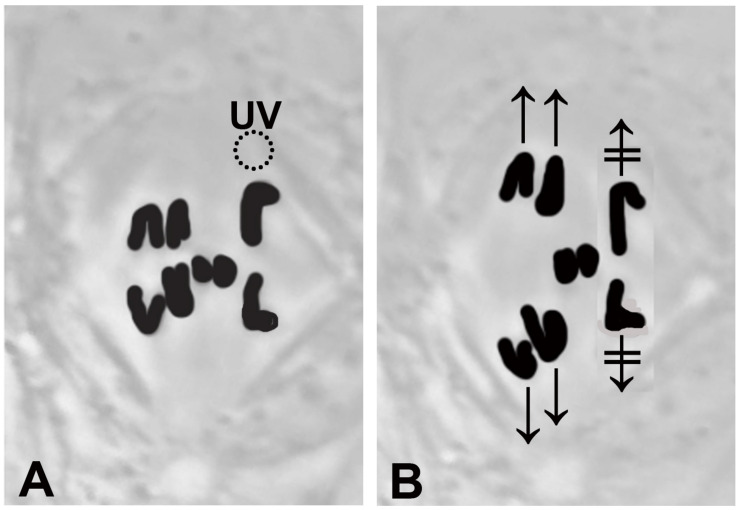
A drawing that illustrates stoppage of anaphase chromosomes after a single kinetochore fibre is irradiated with a UV microbeam. **(A)** Chromosomes appear black. The three disjoining autosome pairs have started moving to the poles, and the two smaller sex chromosomes remain at the equator. The dashed circle, the position of the UV microbeam, irradiates the spindle fibre of the chromosome to the upper right. **(B)** After the irradiation neither the upper right chromosome nor its partner (lower right chromosome) have moved (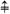 and 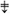). The chromosomes in the other two pairs have continued moving normally.

When irradiation of one kinetochore fibre stopped the anaphase movement of the associated chromosome, the partner chromosome moving to the opposite pole also stopped moving (Forer, 1966; Sillers and Forer, 1981a, b; Adames and Forer, 1996; Yin and Forer, 1996; Ilagan and Forer, 1997); the other chromosomes were not affected, as illustrated in Figure 1. UV microbeam irradiation of the interzone uncoupled the separating chromosomes in that only the chromosome attached to the irradiated spindle fibre stopped moving, not its partner (Yin and Forer, 1996). This does not necessarily implicate tethers though, because we do not know if the UV irradiation damaged tethers. To test whether tethers are required for coupling the movements of separating partner chromosomes one needs to determine if the tethers have been affected. We can deduce that we have severed tethers by using a visible-light laser to first sever an arm and then to irradiate between the arm fragment and the partner telomere to stop the motion of the arm fragment (e.g., Figure 4 of Sheykhani et al., 2017). However, we cannot use a visible light laser both to cut tethers and to stop chromosome movement: visible-light laser microbeam irradiations sever kinetochore fibre microtubules in crane-fly spermatocytes (Forer et al., 2013, 2018; Sheykhani et al., 2017) in grasshopper spermatocytes (Chen and Zhang, 2004) and in PtK cells (Elting et al., 2014) but they do not stop anaphase chromosomes from moving. Thus we cannot use visible-light laser irradiation to do this experiment.

**FIGURE 2:**
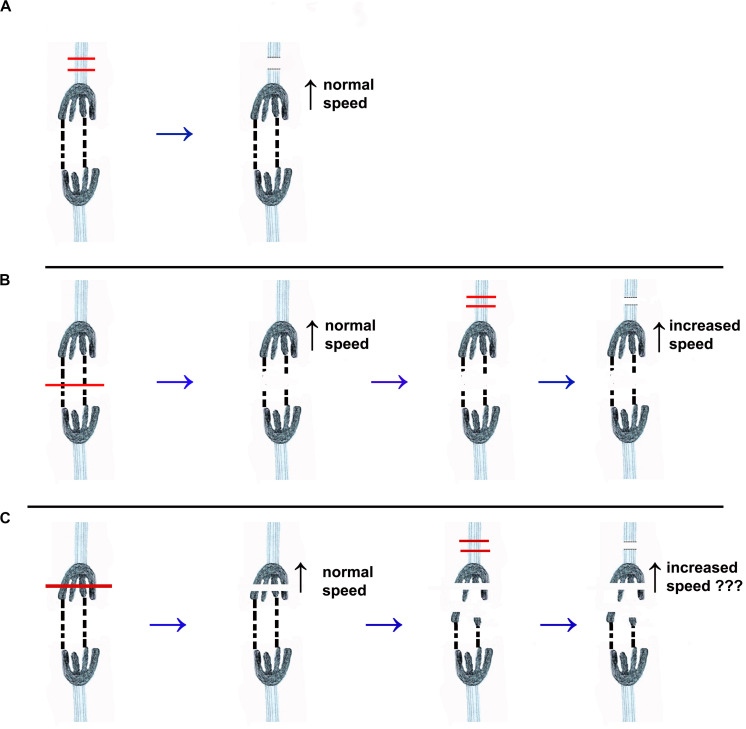
Drawings that illustrate the different experimental protocols discussed in the text. One pair of separating chromosomes is illustrated. The chromosomes are dark, with four trailing arms. Tethers that connect two of their arms are represented by dashed black lines. The kinetochore spindle fibres are represented by light blue sets of lines extending from each kinetochore. **(A)** The red lines illustrate the cutting by laser or UV microbeam of the kinetochore spindle fibre associated with the top chromosome. After the microtubules are severed both chromosomes move with normal (pre-irradiation) speed. (UV microbeam: Forer, 1966; Spurck et al., 1997; Laser: Sheykhani et al., 2017; Forer, unpublished). **(B)** Illustrates the same cutting of the kinetochore spindle fibre, using a laser, after the tethers are cut (at the position of the red line across the tethers in the first image). Cutting the tethers does not change the velocity of either chromosome. After its kinetochore spindle fibre was severed the velocity of the upper chromosome increased (Sheykhani et al., 2017) while the partner chromosome moved at pre-irradiation speed. **(C)** Illustrates the protocol in the experiments we present herein. The arms are severed first (red lines in the first image), such that the tethers no longer mechanically connect the two kinetochores. One of the kinetochore spindle fibres is then severed, and we then determine whether the associated chromosome speeds up.

There is another phenomenon in which the movements of separating chromosomes are coupled, however, for which we *can* use visible-light laser irradiations to test the possible role of tethers. That phenomenon is the continued movements of anaphase chromosomes after their kinetochore microtubules are severed. In crane-fly spermatocytes, anaphase chromosome movement is not altered when kinetochore fibre microtubules are severed by visible-light laser irradiations (Sheykhani et al., 2017; Forer, unpublished): both the attached chromosome and its partner chromosome move poleward with their original speeds (Figure 2A), similar to after UV-microbeam irradiations (Sillers and Forer, 1983; Spurck et al., 1997). In other cell types, however, the chromosomes attached to the severed kinetochore microtubules *accelerate* towards their poles after kinetochore microtubules are severed while the partner chromosomes continue to move at normal speeds. This holds for UV microbeam irradiations in newt cell fibroblasts (Spurck et al., 1997) and for visible-light laser microbeam irradiations in grasshopper spermatocytes (Chen and Zhang, 2004) and in PtK cells (Elting et al., 2014). We previously tested whether tethers coordinate movements of separating partner chromosomes in crane-fly spermatocytes using as criterion that chromosomes accelerate after their kinetochore microtubules are severed. When a visible-light laser was used first to sever tethers between separating chromosomes, and then to sever the kinetochore spindle fibre microtubules, the chromosome associated with the severed kinetochore microtubules accelerated while the partner did not (Sheykhani et al., 2017), as diagrammed in Figure 2B, accelerating exactly as described in newt, PtK and grasshopper cells (Spurck et al., 1997; Chen and Zhang, 2004; Elting et al., 2014). Since severing the tether removes the “prohibition” on acceleration, this result suggests that tethers that connect separating telomeres are involved in regulating speeds of separating chromosomes. This important conclusion has a possible flaw, though, because laser irradiation of the interzone that severs the tethers may also cause collateral damage to other interzonal components and these other components may coordinate the movements of the partner chromosomes.

In the present experiments we disabled tethers without cutting the interzone, thereby eliminating the possible objection to the earlier conclusions. The tethers were disabled by severing arms from anaphase chromosomes (illustrated in Figure 2C). This eliminates the mechanical link and the tension between the separating chromosomes by freeing the arm fragments from one of the chromosomes. After disabling tethers in this way, we then cut the kinetochore spindle fibre microtubules. The associated chromosomes accelerated after their kinetochore fibres were severed, implicating tethers in the regulation of poleward speeds of separating anaphase chromosomes in crane-fly spermatocytes.

## Materials and Methods

We studied meiosis-I spermatocytes from crane flies (*Nephrotoma suturalis* Loew) reared in the laboratory using methods described elsewhere (Forer, 1982). The experimental methods used in this study are described in detail in Sheykhani et al. (2017). In brief, larvae of the proper stage were covered with Halocarbon oil, the testes were removed, and contents of individual testes were spread on a coverslip in Ringers solution that contained fibrinogen. Thrombin was added to form a clot to hold the cells in place, the coverslip was placed in a perfusion chamber, and the cells were perfused with insect Ringers solution. Cells were observed using phase-contrast microscopy (63×, NA 1.4, Zeiss Plan Apochromatic lens) and digital images were recorded every 2–4 s. Laser irradiations were with a 200 fs pulsed laser, wavelength 740 nm, using the laser microscope described in detail elsewhere (Shi et al., 2012; Harsono et al., 2013). Before each experiment, the aiming accuracy of the focused laser beam was adjusted by targeting individual dried blood cells on a microscope coverslip so that the laser lesions (in the blood cell) were coincident with the target lines projected on the video monitor, as illustrated in Berns (2020). At near the energy threshold for production of a circular hole in an individual blood cell, the laser plane of focus is quite tight: if the blood cells are slightly out of focus, the laser irradiation does not produce a small circular spot, thus indicating that the beam is being focused slightly above or below the focal plane of the target cell. Because the red blood cells must be extremely flattened to act as a suitable target, and because not all red blood cells in any given blood smear are flat enough, we often used as targets the dried blood serum that surrounds the less-flattened red blood cells. Because the laser has a very tight plane of focus, in viewing cells we try to restrict our experiments to cells flattened on the coverslip. If the target is too far above the coverslip, the dispersion that occurs because of the mismatch of refractive indices between cell and coverslip causes a shift in focus of the beam. When that occurs, chromosomes we aim at sometimes are not cut but those in a different focus level may be. In irradiating cells, we try to keep the laser power to the minimum that we need to sever chromosomes and tethers because when the power is too high the irradiation can blow up the cell. Prior to experimentation we judge the power to use via test cuts of chromosomes. Since the plane of focus of the laser is narrow, to cut thicker objects we cut the same position (seen in the two-dimensional image of the cell) in different focal levels. For example, for chromosome arms that are ∼1 μm thick, a line was drawn on the computer screen across the image of the arm (or arms) and then the laser was fired to cut in either 3 or 5 *Z*-axis planes, separated by 0.4 μm. Spindle fibres were cut similarly, in 3 *Z*-axis planes.

Once the sequences were recorded, images from the experiments were cropped using Irfanview freeware and time-stamped using data contained in the individual image (png) files; the resultant bmp images were converted to avi files using VirtualDub2 freeware. Graphs of distance versus time were obtained from the avi files using an in-house program (Wong and Forer, 2003) that measures distances between user-specified points. We manually specified the points to be measured (e.g., telomeres and/or kinetochores) on the individual images and the program measured the distances of those points from a fixed point that we specified at a pole or at the equator.

## Results

Crane-fly spermatocyte meiosis-I has been described previously (Forer, 1980). Briefly, 2*n* = 8 and each spermatocyte has three bivalent and two univalent chromosomes. At anaphase the bivalents disjoin and move to the stationary poles but the univalent sex-chromosomes (X and Y) remain at the equator (Figure 1); the sex chromosomes do not move towards the poles until the autosomes have reached the poles and the spindles start to elongate. Throughout anaphase each univalent is attached to two kinetochore spindle fibres, one connected to each pole. When the two univalents segregate to opposite poles, for each univalent one of its spindle fibres shortens and the other elongates.

The experiment requires that chromosome arms that have tethers are completely severed, to eliminate mechanical connections between separating chromosomes. In theory one can identify the two arms that are attached to tethers by severing arms one at a time until arm fragments from the two tethered arms move across the equator to the partner telomere. But because tethers lose elasticity as anaphase proceeds one cannot always rely on movement of arm fragments to identify the two tethered arms. When arm fragments did not move, we severed all four arms, to ensure that we disabled the tethers.

**FIGURE 3:**
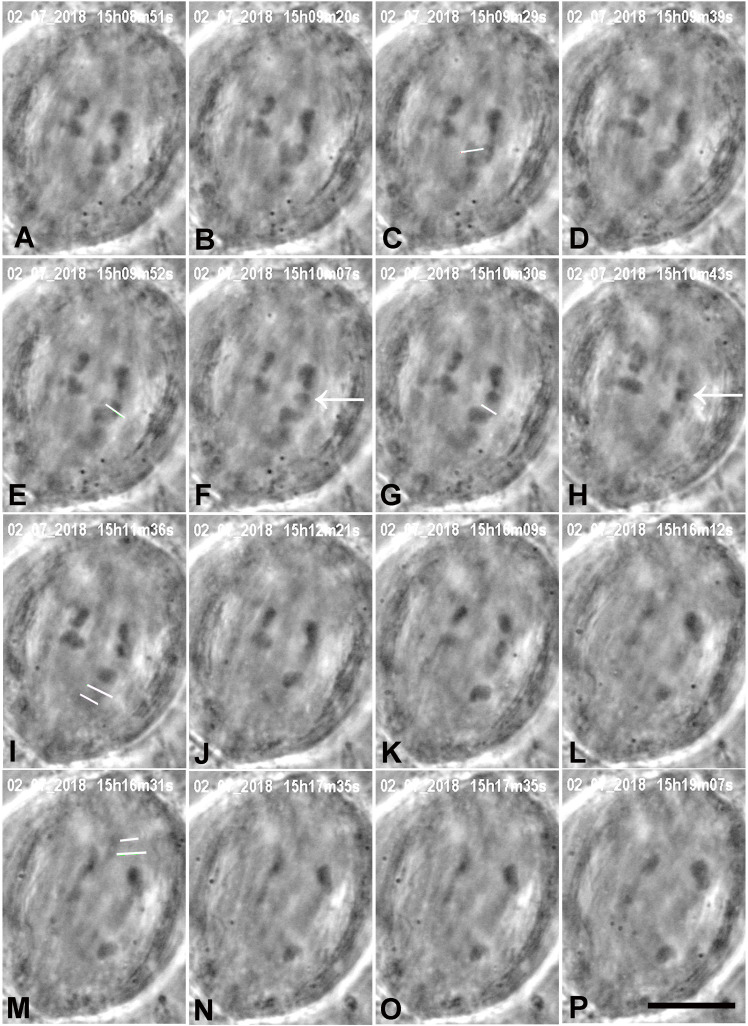
A cell in which several laser cuts were required to sever all tethered arms. **(A)** Anaphase. The first cut (white line in **C**) severs the arm (cf. **B,D**); the arm fragment (not visible) quickly moved to its partner chromosome. The second laser cut (white line in **E**) produced an arm fragment (arrow in **F**), and after a second laser cut (white line in **G**) the arm fragment (arrow in **H**) moved to its partner chromosome **(H)**. We cut kinetochore fibres of both the chromosome with the amputated arms and its partner moving to the opposite pole. The two white lines in **I** indicate the positions in which the kinetochore spindle fibre was cut (shortly after this image). **(J–L)** The lower right chromosome accelerated after **I**, as seen in the movement graph of this cell shown in Figure 10. The two white lines in **M** indicate the positions at which the kinetochore fibre of the partner chromosome was cut. **(N–P)** the upper right chromosome accelerated after **M**, as shown in Figure 10. The times in this (and all subsequent figures) are in h:min:s as time-stamped in the digital images. The black line at the bottom of image **P** represents 10 μm.

**FIGURE 4:**
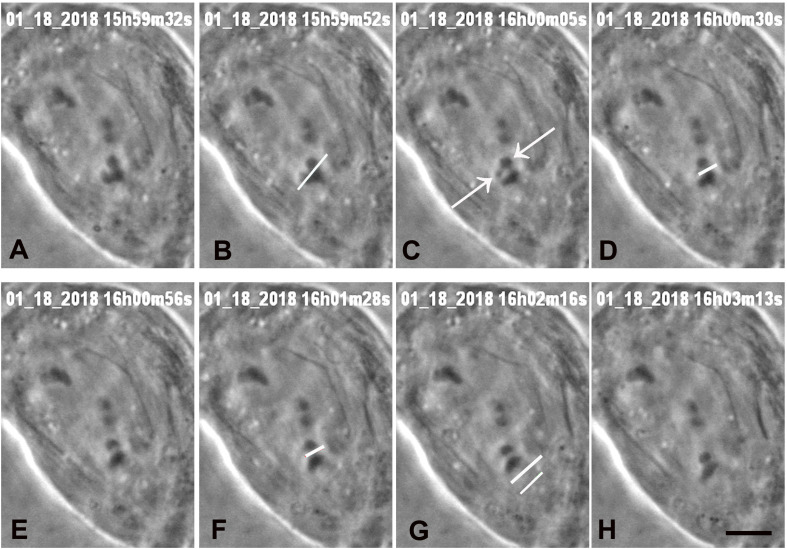
A cell in which three laser cuts were used to sever all arms from one chromosome. **(A)** Anaphase, prior to laser irradiation. The arms of the lower chromosome were cut three times, at positions indicated by the white lines in images **(B,D,F)**, all shown just as the laser was cutting the arms. Two arm fragments produced by the cut are indicated by arrows in **C**. The cut shown in **D,F** were at the same position, to ensure that the arm fragments were completely severed from the chromosome. The two lines in **G** indicate the positions that the laser cut the kinetochore spindle fibre, shown just before the laser cut the fibre. **(H)** The chromosome associated with the cut kinetochore fibre accelerated after the cut, as seen in Figure 7, a movement graph of this cell. The scale bar in image **H** represents 5 μm.

Another complication arises because sometimes arms are not completely severed. In previous experiments, arms were ostensibly severed, the arm fragments rapidly moved towards their partner telomeres, the tethers were severed, and while severing the tethers caused *most* arm fragments to stop moving, sometimes the moving arm fragment reversed direction and moved back to its original arm (Figure 5 of Sheykhani et al., 2017). This indicates that between some arm fragments and their original chromosomes there are “remnant” chromosomal connections that we cannot see. The remnant connection is much weaker in elasticity than the tether, because the arm fragment moves rapidly towards its partner even when incompletely severed, but the remnant must be strong enough to pull the arm fragment back to the chromosome arm when the tether is cut. The complication for our present experiments is clear: we rely on cutting the arm to sever its mechanical connection to the chromosome but in some cases the arm fragment remains mechanically attached, albeit weakly. Our experimental logic in disabling tethers requires that the separating chromosomes *not* be mechanically attached. To deal with this complication we often repeated cuts at the original site where the fragment was severed, to ensure that the arm fragment was completely disconnected from the rest of the chromosome (Figures 3, 4). Further, we usually cut kinetochore fibres associated with the chromosomes from which the arm fragments were amputated. We did this because we thought that any remnant connections that might remain between the arm fragment and the original chromosome would have considerably less force than the tether and might not register as mechanical linkage between the separating chromosomes whereas the intact tether on the other chromosome might still register the tension.

**FIGURE 5:**
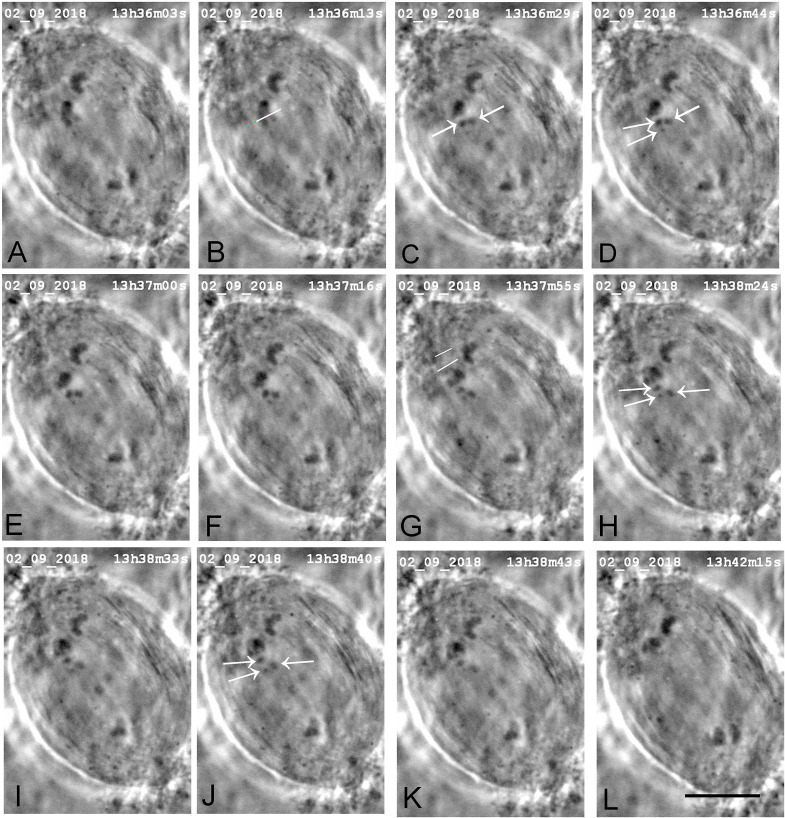
A cell in which one laser cut severed all tethered-arms from one chromosome. **(A)** Mid-anaphase. The arms were severed from the upper left chromosome. The white line in image **B** indicates the line of the laser cut, shown just before severing the arms. Two chromosome arm fragments are seen in image **C** (arrows) and three are indicated by arrows in **D,H,J**, two of which seemed to move a little towards their partners; the third did not. The two lines in image **G** indicate the positions on the kinetochore spindle fibre that the laser cut, shown just before the cutting. **(H–L)** the chromosome associated with the irradiated kinetochore fibre in G accelerated after the laser cut, as shown in Figure 9, which is a movement graph of this cell. The scale bar in **L** represents 10 μm.

**FIGURE 6:**
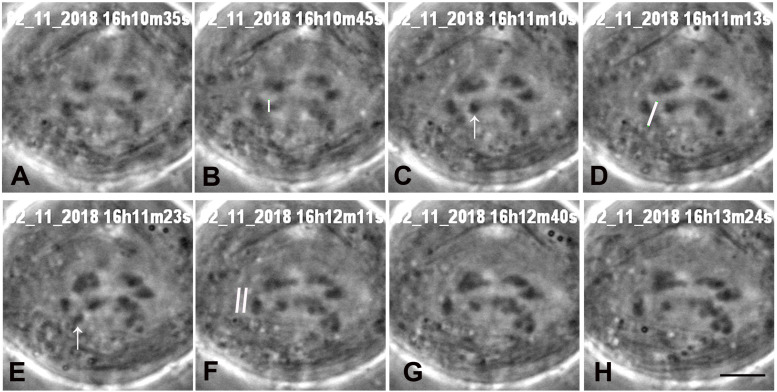
A cell in which two laser cuts were needed. **(A)** Early anaphase. One laser cut severed one arm (at the position of the white line in **B**, shown just before the cut). The arrow in **C** points to the arm fragment. Another laser cut severed another arm (the white line in **D**, just before the cut). The arrow in **(E)** points to the second arm fragment. The two white lines in **F** indicate the position at which the kinetochore fibre was cut. Both fragments moved towards their partner telomeres. **(G,H)** the chromosome associated with the cut kinetochore fibre accelerated after **F**, as seen in Figure 8, a graph of movements in the same cell. The black line in **H** represents 5 μm.

We successfully severed all tethered-arms from single chromosomes in 21 cells. After the telomere-containing arm fragments were no longer attached to the rest of the chromosomes we severed the kinetochore spindle fibres of the associated chromosomes, as illustrated in the montages in Figures 3–6. In some experiments we needed only one cut across several arms (Figure 5); in others, several cuts were necessary because of the configurations of the arms, as illustrated in Figures 3, 6, for two or three cuts across trailing arms. Not all laser-cut arm fragments moved across the equator to the partner telomere or even moved towards the partner telomere, depending on which of the four arms was cut and depending on whether the tethers were elastic. Arm fragments did not move from the severed positions in cells later in anaphase (Figures 4, 5), but did move towards (or to) their partners in cells earlier in anaphase (Figures 3, 6). As can be seen in these montages, in addition to cutting the chromosomes, the laser also cut laterally outside chromosome arms. The cuts also extend underneath or above the arms since the laser line was moved to several *Z*-axis planes when cutting arms. But we never cut in the interzonal region between telomeres.

**FIGURE 7:**
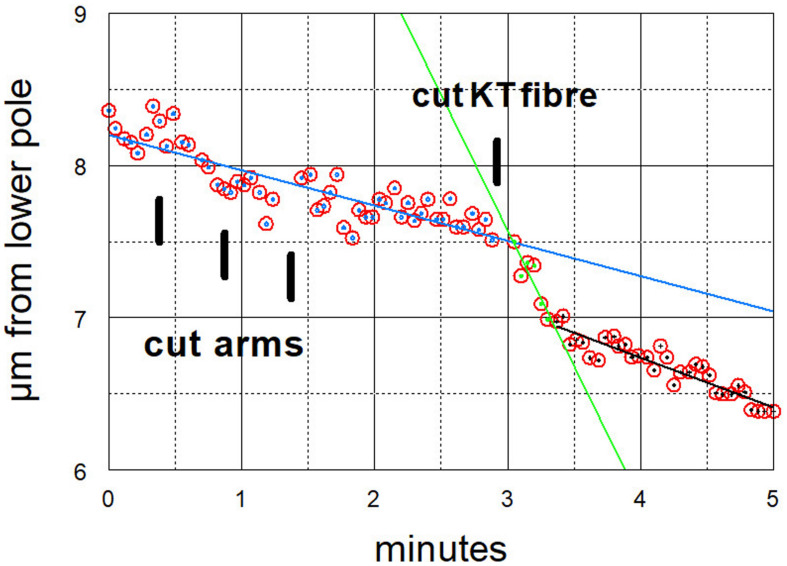
Graphical representation of the movements of one chromosome (Images of the cell are presented in Figure 4). The arms were cut three times (at just before 30 s, just before 60 s, and just before 90 s) and then at just before 3 min its kinetochore fibre was cut (labelled ‘cut KT fibre’). The positions of the kinetochore were measured as distance from the bottom pole (a fixed position). The chromosome accelerated immediately after its kinetochore fibre was cut; it slowed down after about 20 s to near its previous speed. The lines in this and all subsequent graphs are lines-of-best-fit as calculated by the computer program. For this chromosome the pre-irradiation velocity was 0.23 μm/min (blue line), the acceleration velocity was 1.8 μm/min (green line), and the post-slowdown velocity was 0.33 μm/min (black line).

**FIGURE 8:**
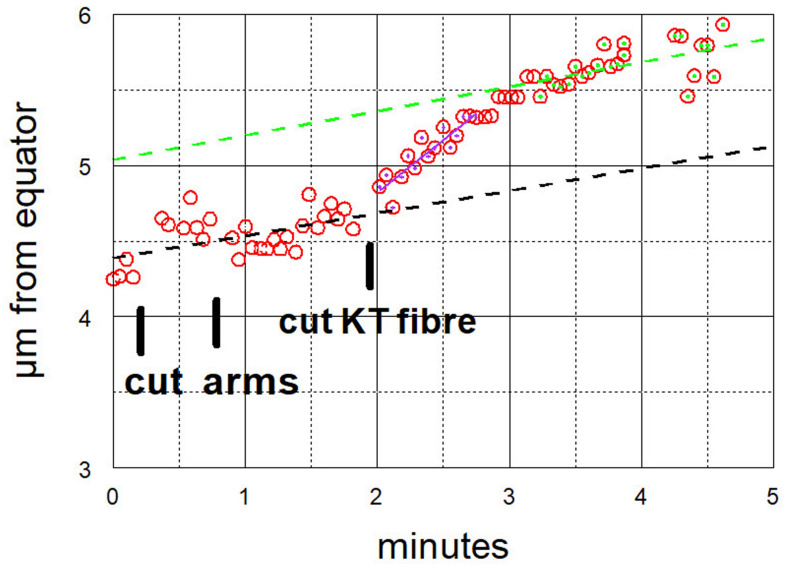
Graphical representation of the movements of one chromosome after its arms were cut twice, and then its kinetochore fibre was cut (labelled ‘cut KT fibre’) (Images of the cell are presented in Figure 6). The positions of the kinetochore were measured as distance from a fixed point at the equator. The chromosome accelerated immediately after its kinetochore fibre was cut; it slowed down after about 1 min to near its previous speed. For this chromosome, the pre-irradiation velocity was 0.15 μm/min (dashed black line), the acceleration velocity was 0.70 μm/min (magenta), and the post-slowdown velocity was 0.16 μm/min (green).

**FIGURE 9:**
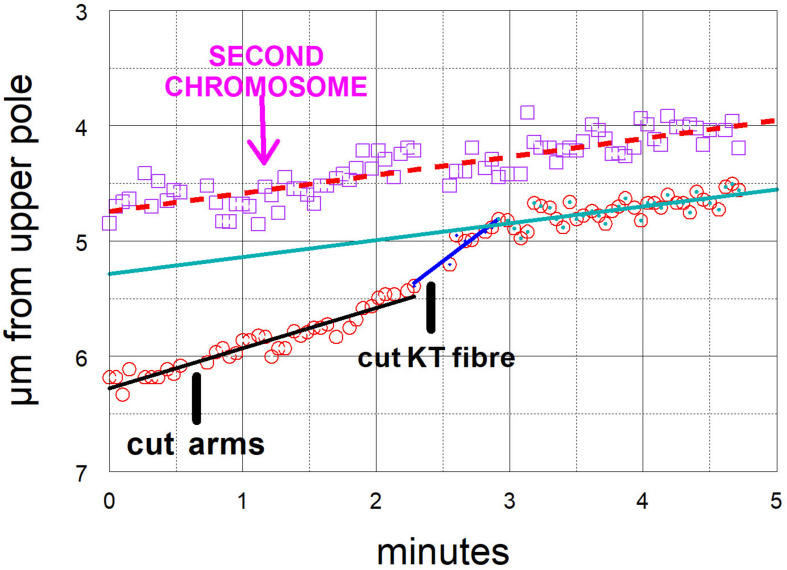
Graphical representations of the movements of two chromosomes moving to the same pole, one whose kinetochore fibre was cut (at ‘cut KT fibre’) and the other (labelled “second chromosome”) whose fibre was not cut. (Images of the cell are presented in Figure 5.) The kinetochore positions were measured as distance from the upper pole (a fixed point). The chromosome whose kinetochore fibre was cut accelerated immediately after the cut and slowed down after about 30 s to a speed slower than its original speed, but one close to that of the second chromosome, whose movement was not changed by any of the laser cuts. For this chromosome the initial velocity was 0.35 μm/min (black dashed line), the acceleration velocity was 0.88 μm/min (blue line), and the post-slowdown velocity was 0.15 μm/min (green line). The velocity of the other chromosome was 0.16 μm/min (dashed red line).

**FIGURE 10:**
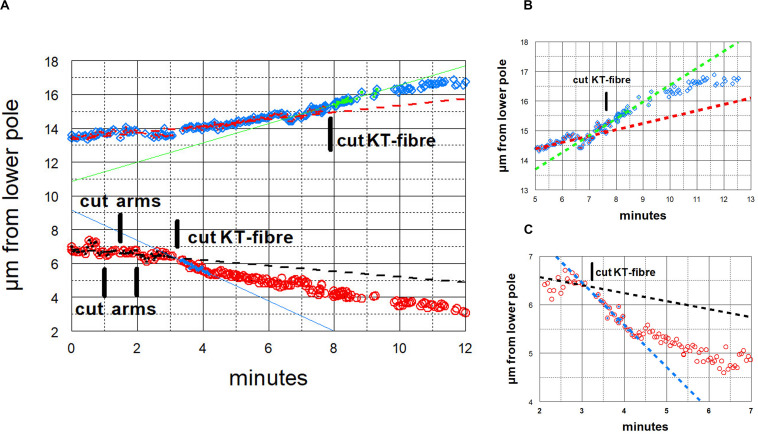
Graphical representations of the movements of a chromosome and its partner moving to the opposite pole. (Images of the cell are presented in Figure 3.) Panel **(A)** illustrates the overview, and panels **(B,C)** show the same points in more detail, focusing on the accelerations after cutting the kinetochore fibres of the two chromosomes, panel **(B)** for the chromosome moving to the upper pole and panel **(C)** for the chromosome moving to the lower pole. After chromosome arms were cut on the chromosome moving to the lower pole, that chromosome’s kinetochore fibre was cut (at ‘cut-KT-fibre’), immediately after which the chromosome accelerated. After about 60 s it slowed down to near its initial speed. Later the kinetochore fibre was cut on the partner chromosome moving to the opposite (upper) pole. That chromosome immediately accelerated and after about 90 s it slowed down to near its initial speed. The initial velocity of the bottom chromosome was 0.17 μm/min (dashed black line), and its accelerated velocity was 0.90 μm/min (blue line); the initial velocity of the upper chromosome was 0.20 μm/min (dashed red line), and its accelerated velocity was 0.57 μm/min (green line).

**FIGURE 11:**
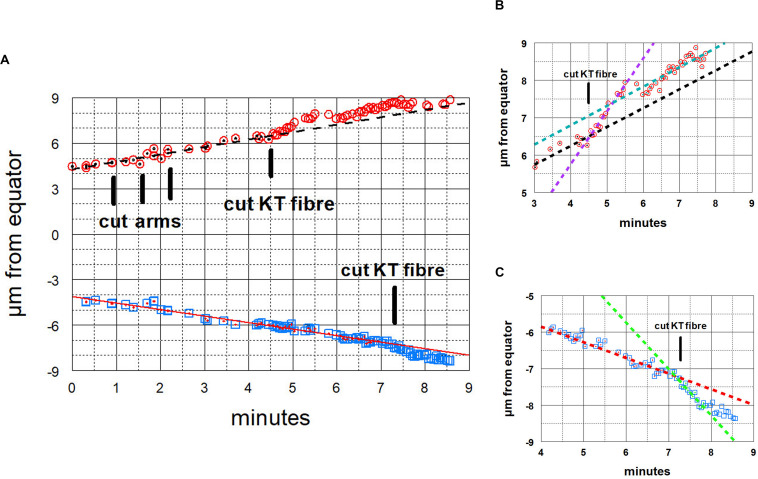
Graphical representations of the movements of a chromosome and its partner moving to the opposite pole. Panel **(A)** illustrates the overview, and panels **(B,C)** show the same points in more detail, focusing on the accelerations after the kinetochore fibres of the two chromosomes were cut, panel **(B)** for the chromosome moving to the upper pole and Panel **(C)** for the chromosome moving to the lower pole. After chromosome arms were cut on the chromosome moving to the upper pole, that chromosome’s kinetochore fibre was cut (at the time labelled ‘cut KT fibre’), immediately after which the chromosome accelerated. After about 60 s it slowed down to near its initial speed. Later the kinetochore fibre was cut on the partner chromosome moving to the opposite (lower) pole, after which that chromosome immediately accelerated; after about 45 s it slowed down to near its initial speed. The initial velocity of the upper chromosome was 0.51 μm/min (dashed black line), its accelerated velocity was 1.4 μm/min (dashed magenta line in **B**), and its post slowdown velocity was 0.52 μm/min (dashed green line in **B**). The initial velocity of the lower chromosome was 0.43 μm/min (red line) and its accelerated velocity was 1.3 μm/min (dashed green line in **C**).

Immediately after cutting the spindle fibres the chromosomes associated with the cut kinetochore fibres accelerated (moved faster than previously) for between 20 and 100 s, and then returned to a slower speed, comparable to the starting speed, as illustrated graphically in Figures 7–9. Though in most of our experiments we cut the kinetochore fibres of the chromosomes with amputated arms, in some cells we also cut the kinetochore fibre of the opposite chromosome, first the kinetochore fibres associated with the chromosomes with amputated arms and later the kinetochore fibres of the chromosomes moving to the opposite poles. Both cuts resulted in the associated chromosomes moving faster; two examples are shown in Figures 10, 11. Both chromosomes accelerated even in 1 cell in which the opposite kinetochore fibres were cut simultaneously. Thus, after disabling the tethers by mechanically disconnecting partner chromosomes, either partner chromosome accelerated when its kinetochore fibre was cut.

**FIGURE 12:**
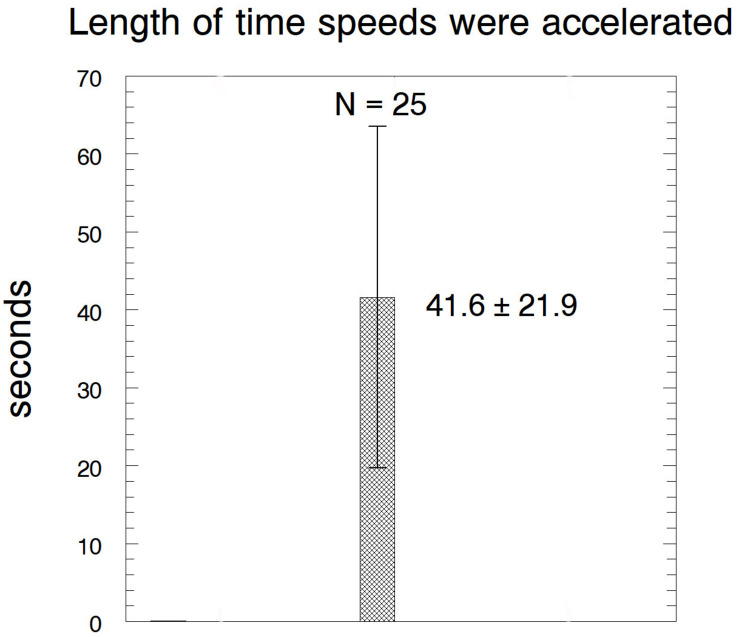
Bar graph showing the lengths of time that the chromosomes remained at accelerated speeds after their kinetochore fibres were cut, showing average ± standard deviation, and the number of chromosomes measured.

**FIGURE 13:**
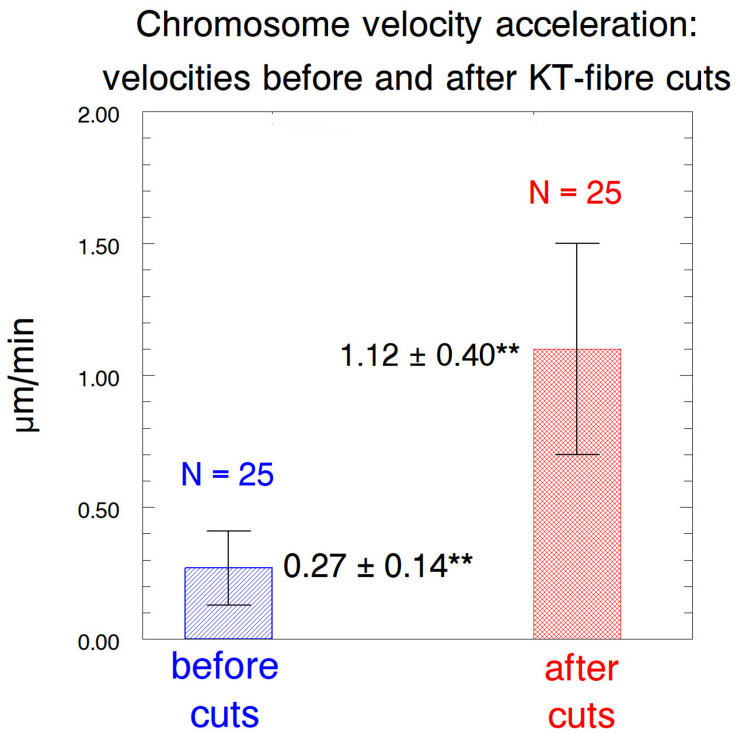
Bar graph comparing the chromosome speeds before and after their kinetochore fibres were severed, showing average values ± standard deviations, and numbers of chromosomes measured. **Indicates significant differences: using Students *t*-test the chance that the averages are from the same distribution is *p* < 0.00001.

After cutting kinetochore fibres the acceleration period lasted on average ∼41 s before the chromosomes slowed down to near their original speeds (Figure 12). The average accelerated anaphase velocities for individual chromosomes were ∼4× the pre-irradiation velocities (Figure 13).

## Discussion

A main conclusion from our experiments is that tethers coordinate the movements of separating anaphase chromosomes in crane-fly spermatocytes. Anaphase chromosomes with normal tethers do not speed up when their kinetochore spindle fibres are severed but chromosomes with cut tethers do (Sheykhani et al., 2017). To exclude the possibility that damage to non-tether interzonal components uncoupled the movements, we disconnected the separating chromosomes by severing the tethered arms from individual chromosomes, after which the chromosome associated with a cut kinetochore fibre accelerated while the partner did not. Therefore, tethers are responsible for, or at least are required for, regulating the speeds of separating partner chromosomes. Extending this conclusion, we assume that when both partners stop moving after UV microbeam irradiation of only one kinetochore fibre (Figure 1; Forer, 1966; Sillers and Forer, 1981a, b; Adames and Forer, 1996; Yin and Forer, 1996; Ilagan and Forer, 1997) the stoppage of the partner chromosome (moving to the opposite pole) is due to tethers. Consistent with this is that UV microbeam irradiation of the interzone uncouples the movements of the separating anaphase chromosomes: after first irradiating the interzone, the chromosome associated with the irradiated kinetochore spindle fibre stops moving – the partner moves normally (Yin and Forer, 1996). Crane-fly spermatocytes are not the only cells with coordinated movements of separating anaphase chromosomes. In grasshopper spermatocytes chromosome velocity varies with temperature, and when one half spindle was heated to a higher temperature than the other, the separating chromosomes moved poleward with the same speeds, that speed governed by the temperature of the cooler half spindle (Nicklas, 1979). It could be that tethers influence chromosome coordination in the grasshopper cells also.

How might tethers coordinate the separating chromosomes? One possibility is that the tension on chromosome arms [stretching them by about 10% (Forer et al., 2017)] is transmitted to the kinetochores, similar to effects of stretching chromosome arms described previously. Nicklas and Koch (1969), using micromanipulation needles to stretch the arms, showed that meiotic bivalents reorient when the arms are not under tension; that tension on chromosome arms stabilises attachments to the spindle, even faulty ones (Nicklas and Ward, 1994) and that tension at the kinetochore affects kinetochore phosphorylation levels (King and Nicklas, 2000) and influences checkpoint signals (Nicklas et al., 1995). Tension affects these and other processes because of phosphorylation of kinetochore proteins via Aurora kinase and recruitment of checkpoint proteins to the kinetochore (Skoufias et al., 2007; Saitoh et al., 2008; Liu et al., 2009; Wang et al., 2012). Given these well-documented effects of tension on kinetochores, it is possible that tension from tethers might affect kinetochores, which in turn could regulate chromosome velocities. One uncertainty in this argument, though, relates to the small forces produced by tethers relative both to forces that propel chromosomes poleward, and to forces applied by the micromanipulation needle to stretch the chromosomes. Since cutting tethers does not change anaphase chromosome velocities (Sheykhani et al., 2017; Figures 9–11), tether forces are considerably smaller than the forces propelling the chromosomes poleward, say ≤10% of the poleward forces; while in most of the experiments we do not know the forces applied to chromosome arms when stretching them with the microneedle, in some of the experiments the forces applied were estimated to be three times that needed to move chromosomes poleward (King and Nicklas, 2000). Tethers do not produce anywhere near those forces, but it is conceivable that even very low forces on chromosome arms might affect kinetochore interactions, which then would regulate the speed of the attached chromosomes.

Another possibility is that tensegral interactions might influence the movements of chromosome partners. Tensegrity involves stabilised interactions between networks of tension-producing components and networks of load-bearing components (struts). Tensegrity has been considered a major foundation of the structure of living cells: many changes in cell function and structure, both overall and local, including position-specific mechanical-transduction, can be explained assuming that cell structure is based on tensegrity, i.e., that cells contain an interacting system of tensile elements and struts (Ingber, 1993, 1997, 2003a,b). A variety of cell configurations and functions of the cytoskeleton can be explained on this basis, including mechanisms of mitosis (Pickett-Heaps et al., 1997) and possible mechanisms by which chromosomes in interphase and mitotic spindles remain in a fixed order (Nagele et al., 1995, 1998, 1999), as considered by Maniotis et al. (1997). Tensegral systems are capable of transmitting local disturbances to other positions (Ingber et al., 1994, 2000; Ingber, 2003a, b), so it is possible that removal of one of the tensile structures (tethers) in an integrated architectural network of compression and tension-producing elements can regulate force-producing components that cause chromosome movement.

Do our experiments unequivocally prove that tethers coordinate chromosome movements or could there be alternate interpretations of our data? Our irradiations unavoidably included spindle regions immediately adjacent to chromosome arms so we cannot rule out the possibility that other spindle components were damaged by the laser. However, any alternative interpretation must satisfy several conditions. One is that the component responsible for coordinating the movements must be in the interzone, between the arms of separating chromosome: this is because chromosome movements become uncoupled when that region is cut with the laser (Sheykhani et al., 2017). A second condition is that the alternative component must be adjacent to separating arms throughout anaphase: this is because of the present experiments in which we severed the arms. A third condition is that the component must not extend in front of the kinetochore: this is because cuts in front of the kinetochore do not uncouple the movements. While it is conceivable that there are components such as “non-kinetochore” microtubules that might extend through the interzone and around both sets of chromosome arms, and that they do not extend past the kinetochores, and that they continue to elongate throughout anaphase (during which time the spindles do not elongate), we think it is far more likely that the tethers are the communication links, acting via the tension they produce between the separating chromosomes. This seems likely because we do not know of any data that show that non-kinetochore microtubules satisfy the three conditions, and because cutting arms from chromosomes minimally impacts the non-kinetochore microtubules, if at all, as follows.

Chromosomes accelerate after first tethers and then kinetochore fibres are severed (Sheykhani et al., 2017); later they slow down again. The accelerated period lasts about three times longer than after arms and kinetochore fibres are severed (Table 1, column 3, rows 1 and 2). We suggest that this is because cuts in the interzone (when cutting tethers) depolymerise non-kinetochore microtubules whereas cuts to arms do not. We assume that accelerated movements are slowed down when the chromosome or its kinetochore stub encounter obstacles such as non-kinetochore microtubules, as suggested by Spurck et al. (1997) and Chen and Zhang (2004). With fewer non-kinetochore microtubules to act as obstacles for poleward motion, the accelerated movements last longer. Consistent with this, non-kinetochore microtubules are more labile than kinetochore microtubules to cold (Salmon and Begg, 1980; Schaap and Forer, 1984) to depolymerisation by nocodazole (Cassimeris et al., 1990) and, most relevant to this discussion, are labile to spindle irradiation with UV microbeam (Czaban and Forer, 1994) or with visible-light laser irradiation (Elting et al., 2014). To test whether microtubule obstacles interfere with accelerated chromosomes one can compare the same experiment – cutting tethers and then cutting kinetochore fibres – performed in two different circumstances, in normal cells versus in taxol-treated cells. Taxol stabilises non-kinetochore microtubules and increases the numbers of microtubules in spindles (Wilson and Forer, 1997; LaFountain et al., 2001), thereby increasing the numbers of obstacles that would end the accelerated movements. As expected, the period of acceleration is three times shorter in taxol treated cells than in control cells (Table 1, column 3, rows 2 and 3), and is the same as that when only arms are severed (Table 1, column 3, rows 1 and 3). This analysis indicates that cutting arms does not have much effect on non-kinetochore microtubules, and thus strongly supports our original interpretation that tethers are necessary for coordinating movements of separating chromosomes.

**TABLE 1:** Comparison between cutting arms *versus* cutting tethers.

	Initial chromosome velocities ± SD	Accelerated chromosome velocities ± SD	Length of acceleration phase ± SD
Cutting only arms (Figures 12, 13)	0.27 ± 0.14 μm/min (*N* = 25)	1.12 ± 0.40 μm/min (*N* = 25)	41.6 ± 21.9 s*,** (*N* = 25)
Cutting tethers (Sheykhani et al., 2017)	0.47 ± 0.21 μm/min (*N* = 19)	0.47 ± 0.21 μm/min (*N* = 19)	125 ± 61 s* (*N* = 19)
Cutting tethers in taxol-treated cells (Forer et al., 2018)	0.15 ± 0.09 μm/min (*N* = 31)	0.87 ± 0.55 μm/min (*N* = 27)	46 ± 19 s** (*N* = 21)

*Statistically significantly different using Students *t*-test (*p* << 0.0005). **Not statistically significantly different using Students *t*-test (*p* ∼0.5).

At present the study of tethers is limited to those able to laser irradiate portions of cells because tethers can be identified only by cutting an arm from a chromosome and seeing if the resultant arm fragment moves to the partner telomere. One could expand the experimental repertoire if one could identify tethers in living or stained cells. The literature from the early 1900s illustrates stained strands that connect the arms of separating anaphase chromosomes and therefore appear to be tethers (Paliulis and Forer, 2018). That the connecting material is different from spindle fibres themselves is especially clear in Figure 11 of Cleland (1926) because the chromosomes in *Oenothera* meiosis are a series of reciprocal translocations (Hejnowicz and Feldman, 2000) with separating anaphase telomeres at an angle to the spindle axis: the stained strands connecting the telomeres in *Oenothera* are also at an angle to the spindle axis and therefore most likely represent tethers. These images suggest that it may be possible to stain for tethers.

Do tethers coordinate the movements of separating anaphase chromosomes in all cells? Some data suggest not. Tethers are present in all animal cells tested so far, including PtK cells (Forer et al., 2017), yet in other cells anaphase chromosomes with severed kinetochore microtubules accelerate even when their tethers are not severed: in PtK cells (Elting et al., 2014) in newt fibroblasts (Spurck et al., 1997) and in grasshopper spermatocytes (Chen and Zhang, 2004). In these cell types, after cutting kinetochore spindle fibres with UV or visible-light laser microbeam irradiation, the associated anaphase chromosomes rapidly moved a short distance backwards (away from the pole), towards their partners (reviewed in Forer et al., 2015). After a very short time they accelerated and moved towards the pole at speeds faster than normal, but after a few minutes they slowed down to their original speed. The fact that the chromosomes moved backwards after their kinetochore fibres were severed from the pole suggests that the tethers were still functional. That the chromosomes then accelerated indicates that the presence of tethers did not prevent the acceleration. This presents a puzzle: why do tethers prevent acceleration in crane-fly spermatocytes but not in PtK cells, newt cells or grasshopper spermatocytes? We can only speculate that either tethers coordinate partner chromosomes in all cells but the manifestation of the coordination is different in cells from different species, or tethers have different functions in cells in different species.

Tangential to the main issue of tether participation in coordinating movements of partner chromosomes is the issue of how anaphase chromosomes move with severed kinetochore microtubules. It seems counter-intuitive that movement should continue, let alone speed up for a short time. The two views on why this occurs have been reviewed in Forer et al. (2015). Briefly summarising the arguments, one group argues that dynein re-locates to the free minus-end of the severed kinetochore microtubules (the kinetochore stub) and then acts with non-kinetochore microtubules to propel the chromosomes poleward (Elting et al., 2014; Sikirzhytski et al., 2014). These authors are silent on why the chromosomes initially move backwards; they invoke a new (different from normal) force-producing mechanism to explain the faster speed; and they are silent on why the movements slow down again. The other group argues that forces that move chromosomes poleward arise from non-microtubule spindle components (Mogessie and Schuh, 2017) perhaps associated with a “spindle matrix” (Pickett-Heaps and Spurck, 1982; Pickett-Heaps et al., 1996; Johansen and Johansen, 2007; Pickett-Heaps and Forer, 2009; Johansen et al., 2011). They argue that kinetochore microtubules act as governors in that microtubule depolymerisation limits the speed of anaphase movement (Nicklas, 1983; Bayles et al., 1993). When kinetochore microtubules are severed, the kinetochore microtubules are no longer connected to the pole, can no longer act as “governors,” and consequently the kinetochore stubs are propelled poleward (by spindle matrix forces) at increased speeds not limited by microtubule depolymerisation. Thus, the same forces that normally propel chromosomes poleward cause the chromosomes to accelerate. This model is silent on why dynein relocates to the tips of severed kinetochore microtubules, but it predicts that the movement slows down when the stub encounters an obstacle (Spurck et al., 1997). The different interpretations remain unresolved, but since chromosomes in *Mesostoma* spermatocytes move poleward very rapidly (up to 200 μm/min) in the absence of kinetochore microtubules (Fegaras and Forer, 2018) and since chromosomes in crane-fly spermatocytes with severed kinetochore microtubules (and severed tethers) accelerate even in the presence of taxol (Forer et al., 2018), it seems likely that forces external to spindle microtubules produce forces for movement. Regardless of which models give correct representations of the poleward forces on chromosomes, models of mitotic spindle forces as a whole must include forces from tethers, forces that act on chromosome arms in the opposite direction to those that propel anaphase chromosomes poleward.

## Conclusion

In conclusion, the experiments reported here indicate that cutting arms from chromosomes (thereby disabling the tethers) disrupts the coordination between separating chromosomes in crane-fly spermatocytes. These data strongly argue that regulation of the speeds of separating anaphase chromosomes in crane-fly spermatocytes is due to the tethers that connect them.

## Data Availability Statement

The datasets generated for this study are available on request to the corresponding author.

## Author Contributions

AF did most of the experimentation, which took place in the laboratory of MB. Both authors contributed to writing the final manuscript.

## Conflict of Interest

The authors declare that the research was conducted in the absence of any commercial or financial relationships that could be construed as a potential conflict of interest.
